# Effects of Soy Isoflavones, Resistant Starch and Antibiotics on Polycystic Ovary Syndrome (PCOS)-Like Features in Letrozole-Treated Rats

**DOI:** 10.3390/nu13113759

**Published:** 2021-10-24

**Authors:** Geethika S. G. Liyanage, Ryo Inoue, Mina Fujitani, Tomoko Ishijima, Taisei Shibutani, Keiko Abe, Taro Kishida, Shinji Okada

**Affiliations:** 1 Food Functionality Laboratory, Graduate School of Agricultural and Life Sciences, The University of Tokyo, 1-1-1, Yayoi, Bunkyo-ku, Tokyo 113-8657, Japan; savimc89@gmail.com (G.S.G.L.); aishiji@mail.ecc.u-tokyo.ac.jp (T.I.); okaokahiroto@gmail.com (T.S.); aka7308@mail.ecc.u-tokyo.ac.jp (K.A.); 2 Laboratory of Animal Science, Department of Applied Biological Sciences, Faculty of Agriculture, Setsunan University, Osaka 573-0101, Japan; ryo.inoue@setsunan.ac.jp; 3 Laboratory of Nutrition Science, Division of Applied Bioscience, Graduate School of Agriculture, Ehime University, Matsuyama 790-8566, Japan; fujitani.mina.uu@ehime-u.ac.jp (M.F.); kishida@agr.ehime-u.ac.jp (T.K.); 4 Kanagawa Institute of Industrial Science and Technology (KISTEC), 3-25-13 Tonomachi, Kawasaki-ku, Kawasaki 210-0821, Japan; 5 Food and Health Sciences Research Centre, Graduate School of Agriculture, Ehime University, Matsuyama 790-8566, Japan

**Keywords:** PCOS, soy isoflavones, resistant starch, antibiotics, gut microbiota

## Abstract

Polycystic ovary syndrome (PCOS) is the most common endocrine disorder in reproductive-aged women. Recently, various dietary interventions have been used extensively as a novel therapy against PCOS. In the present study, we show that soy isoflavone metabolites and resistant starch, together with gut microbiota modulations, were successful in decreasing the severity of PCOS-like reproductive features while increasing the expression of gut barrier markers and butyric acid in the gut. In the letrozole-induced PCOS model rats, the intake of both 0.05% soy isoflavones and 11% resistant starch, even with letrozole treatment, reduced the severity of menstrual irregularity and polycystic ovaries with a high concentration of soy isoflavones and equol in plasma. Antibiotic cocktail treatment suppressed soy isoflavone metabolism in the gut and showed no considerable effects on reducing the PCOS-like symptoms. The mRNA expression level of occludin significantly increased with soy isoflavone and resistant starch combined treatment. Bacterial genera such as *Blautia*, *Dorea* and *Clostridium* were positively correlated with menstrual irregularity under resistant starch intake. Moreover, the concentration of butyric acid was elevated by resistant starch intake. In conclusion, we propose that both dietary interventions and gut microbiota modulations could be effectively used in reducing the severity of PCOS reproductive features.

## 1. Introduction

One of the most common endocrine disorders in women of reproductive age is polycystic ovary syndrome (PCOS). According to the Rotterdam consensus criteria, which is one of the main diagnostic criteria guidelines used worldwide, PCOS diagnosis should include two of the following three characteristics: clinical/biochemical hyperandrogenism, oligo-and/or anovulation and polycystic ovaries on ultrasound [1]. The prevalence of PCOS varies among different ethnicities and depends on the diagnostic criteria used. In general, the worldwide prevalence of PCOS can range between 6 and 18% [2,3,4].

Several risk factors, including obesity, type 2 diabetes, cardiovascular disease and systemic inflammation, are associated with PCOS [3,5]. Moreover, PCOS has also been reported to affect adverse pregnancy outcomes, such as increased miscarriage rates and subfertility by affecting embryonic implantation [6]. PCOS has also been found to alter the lipid profiles in non-obese pregnant women, which may cause frequent adverse pregnancy outcomes [7]. PCOS is a multifaceted disease with diverse etiological factors which makes it difficult to treat [8]. According to the currently available data, PCOS might originate in utero, and PCOS phenotypes may be developed later in life [8]. Ample evidence suggests that genetics play a considerable role in PCOS [9,10]. Additionally, the occurrence of PCOS is enhanced due to various lifestyle, occupational and environmental factors [8,11,12]. Several approaches, such as lifestyle modifications (diet and exercises), insulin-sensitizer therapy, direct hormonal treatments such as contraceptive pills, ovulation induction therapy, etc. are currently being used to treat PCOS [13].

In recent years, diet-based modulations of physiological functions in the human body have gained much attention. Various functional food components have been studied, and they have shown promising modulatory effects on chronic diseases [14]. Nutritional supplements such as omega-3 fatty acids, chromium, selenium, vitamin D, vitamin B, inositol, etc. and herbal medicines such as tea, cinnamon, spearmint, Chinese peony, liquorice, chestberry, etc. have been extensively investigated as novel therapies for use in women with PCOS [15,16,17,18,19,20,21,22,23,24,25,26].

Soy isoflavones, also known as phytoestrogens, are a type of plant-derived polyphenol that have a similar structure to mammalian estrogen and possess weak estrogenic activities [27]. Most soy isoflavones exist as glycosides (daidzin, genistein, glycitin, etc.) and are converted into aglycones (daidzein, genistein, glycitein, etc.) in the small intestine through the action of β-glucosidases of gut bacteria. The nonabsorbed fraction and the re-excreted fraction in the bile are metabolized in the colon by colonic bacteria into the more bioavailable metabolite equol [27,28].

Previous studies on the effects of soy isoflavones on PCOS revealed contradictory results. Some studies have reported that soy isoflavone administration can alleviate PCOS symptoms in animals and PCOS patients [29,30,31,32]. However, a few other studies showed that soy isoflavones can induce PCOS symptoms or that soy isoflavones did not affect the occurrence of PCOS symptoms significantly [33,34,35].

Recent studies show that gut microbiota is linked with the development of metabolic disorders [36,37]. In 2012, Tremellen and Pearce [38] proposed a potential relationship between gut microbiota and PCOS through their dysbiosis of gut microbiota (DOGMA) theory. They stated that diet-induced dysbiosis of gut microbiota can induce leaky gut and that subsequent chronic inflammation increases insulin resistance, which can ultimately induce PCOS symptoms through hyperandrogenism [38]. Since then, several human and animal studies have been performed to investigate the link between gut microbiota and PCOS. Human studies came from countries such as China, Austria, Spain, USA, Finland, Poland, etc. and some of them were related to obesity and insulin resistance in women with PCOS [4,32,39,40,41,42,43,44]. These studies also analyzed the overall composition of gut microbiota and have found changes in bacterial genera in women with PCOS compared to healthy/control individuals [4,40,41,43,44]. Animal studies have been performed using different PCOS animal models [3,4,39,45,46,47,48]. Hence, we observed similarities and dissimilarities in the gut microbial compositions among those studies.

Following the background information, we hypothesized that soy isoflavones and their metabolites that are produced by gut microbiota are key effectors in lowering the severity of PCOS symptoms. We also hypothesized that bacterial invasion into gut mucous through increased gut permeability (leaky gut) is a key effector of PCOS pathogenesis. To examine the effects of soy isoflavone metabolites on PCOS, we increased and decreased the metabolism of soy isoflavones using resistant starch and antibiotics, respectively. Resistant starch is a type of dietary fiber and is fermented by gut bacteria, producing short-chain fatty acids (SCFAs) [49]. The fermentation of resistant starch is thought to contribute significantly to butyrate production in the colon and is dominated by *Ruminococcus bromii* [50,51,52]. Diets enriched with resistant starch have been reported to enhance the abundance of gut bacteria such as *Bifidobacteria* [53,54]. Furthermore, resistant starch has been found to increase the metabolism of soy isoflavones by altering the gut microbiota and enhancing the absorption of metabolites [55,56]. Katsumata et al. (2016) [57] reported that by adding kanamycin to diets, the absorption of equol in daidzein-administered ovariectomized mice decreased. Thus, we used antibiotics to diminish gut microbiota to suppress the metabolism of soy isoflavones.

To investigate our hypotheses, a letrozole-induced PCOS animal model was used. Letrozole is an aromatase inhibitor that is commonly used in PCOS research [3,46,58,59]. Usually, in intervention studies, interventions are performed after the letrozole treatment period [29]. However, a recent study revealed that the removal of letrozole treatment resulted in lower hyperandrogenemia, the resumption of menstrual cyclicity and reversion of the altered gut microbiota back to a normal state [60]. As a solution, Arroyo et al. (2019) [60] proposed that to model PCOS accurately, letrozole treatment should be present during the entire study period. Considering this, in our study, soy isoflavones, resistant starch and antibiotics were given individually and in combination with each other simultaneously with letrozole treatment. This allowed us to analyze their effects on the severity of PCOS diagnostic features. Our animal model displayed all three main PCOS-like reproductive features. Resistant starch and antibiotics successfully altered the metabolism of soy isoflavones. Together, soy isoflavones and resistant starch showed the most promising results in reducing the severity of menstrual irregularity and polycystic ovaries. We also observed notable changes in the overall gut microbiota profiles and leaky gut status in the PCOS rats compared to the control rats. Furthermore, some bacterial genera were found to be associated with PCOS symptoms. Our diet treatments were able to alter the associated gut microbiota directly by reducing the leaky gut status.

## 2. Materials and Methods

### 2.1. Experimental Design

Five-week-old specific pathogen-free (SPF) inbred female Sprague Dawley (SD) rats (*n* = 56) with body weights of 110–130 g were purchased from Charles River Laboratories, Kanagawa, Japan. The animals were caged individually in an animal rearing facility with a temperature of 21 °C–23 °C, a relative humidity of 50–70% and an automated 12:12 h light–dark cycle. All protocols for the animal experiments were approved by the Animal Use Committee of the Faculty of Agriculture at the University of Tokyo (P17-080). The compositions of all the diets used are shown in Table 1. The AIN-93G diet supplied by Oriental Yeast Co. Ltd., Tokyo, Japan was used as the control diet. Soyaflavone HG supplement, which contained 52.1% soy isoflavones (kindly provided by Fuji Oil Company Ltd., Osaka, Japan), and HI-MAZE 260 supplement, which contained 56% resistant starch (kindly provided by Ingredion Inc., Tokyo, Japan), were used in the modulated diets (Supplementary Table S1). The AIN-93G diet was supplemented with Soyaflavone HG to obtain a 0.05% soy isoflavone-based diet. Similarly, the AIN-93G diet was supplemented with HI-MAZE 260 to obtain an 11% resistant starch-based diet.

**Figure 1:**
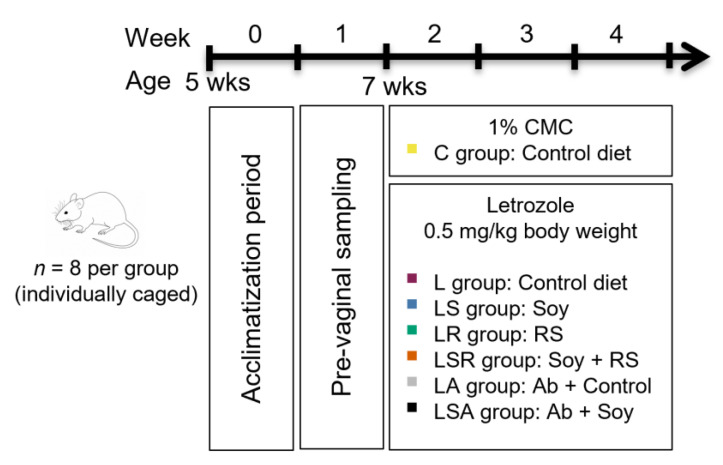
Schematic diagram of the animal experiment. Five-week-old female Sprague Dawley (SD) rats were used for the experiment. After acclimatization and the prevaginal sampling period, rats were divided into seven groups (*n* = 8, 7 weeks old). All animals were housed in individual cages. PCOS symptoms were induced using oral gavage of 0.5 mg/kg letrozole for 21 days simultaneously with the diet treatments. The control group was treated with 1% CMC. Control and L groups were given the control diet. The LS group was given a 0.05% soy isoflavone-based diet. The LR group was given an 11% resistant starch (RS)-based diet. The LSR group was given a mixture of both. The PA group was given the control diet along with an antibiotic cocktail (Ab). The PSA group was given the antibiotic cocktail with the control diet. CMC = carboxymethyl cellulose.

All animals were kept for 7 days for acclimation and then another 8 days for vaginal sampling before the diet treatments. Through the cytological analysis of vaginal smears, it was found that all rats had normal 4–5 day menstrual cycles, so all of them were chosen for further analyses. At the end of the prevaginal sampling period, rats were randomly divided into 7 groups of 8 rats each. The number of animals per group was decided based on a preliminary experiment carried out to investigate the efficacy of letrozole in developing PCOS-like features in rats. In this preliminary experiment, we observed significant PCOS-like reproductive features in the rats and the same method of letrozole induction was used in the current study, as explained later. Animals were 7 weeks old at the start of the treatments, and they were housed in individual cages. The control (C) and PCOS (L) groups were given the control diet. The LS group was given the soy isoflavone-based diet. The LR group was given the resistant starch-based diet. The LSR group was given a mix of soy isoflavone and resistant starch-based diets. The LA group was given an antibiotic cocktail (ampicillin—1 g/L, metronidazole—1 g/L, neomycin—1 g/L, vancomycin—0.5 g/L) and the control diet. The LSA group was given the antibiotic cocktail and the soy isoflavone-based diet. The diet treatments were carried out for 21 days. Simultaneously with the diet treatments, the control group was given 1% carboxymethylcellulose (CMC) solution (supplied by FUJIFILM Wako Pure Chemical Corporation, Osaka, Japan) at a concentration of 2 mL/kg body weight, and the remaining groups were given letrozole (supplied by FUJIFILM Wako Pure Chemical Corporation, Osaka, Japan) at a concentration of 0.5 mg/kg body weight (dissolved in 1% CMC) through oral gavage. After 21 days of diet treatment, all rats were euthanized via exsanguination, and the relevant samples were collected for use in further investigations (Figure 1).

### 2.2. Menstrual Cyclicity Determination through Vaginal Smear Cytological Analysis

Rats were restrained using plastic film tubes called DecapiCones, supplied by Braintree Scientific Inc. MA, USA. A small amount of saline (approximately 0.2 mL) was drawn up into a pipette tip and gently inserted into the vaginal orifice at a depth of approximately 5–10 mm. Saline was flushed into the vagina and out into the pipette 2 or 3 times by gently squeezing and releasing of the bulb of the pipette. A small drop (approximately 10 μL) of the cell suspension was expelled onto a labeled glass slide, and an even thin smear was made. Slides were air dried, stained with Wright–Giemsa stain (modified) supplied by Sigma-Aldrich, St. Louis, MO, USA and observed under an inverted microscope [61,62].

A normal female rat has a 4–5 day menstrual cycle that can be categorized into 4 stages: proestrus, estrus, metestrus and diestrus. These stages mainly consist of 3 cell types: cornified epithelial cells, small and large nucleated epithelial cells and neutrophils (leukocytes). The proestrus stage has many small nucleated epithelial cells. The estrus stage has many cornified epithelial cells and some small and large nucleated epithelial cells. The metestrus stage has many neutrophils and cornified epithelial cells. The diestrus stage also has many neutrophils but none or a few cornified epithelial cells [62]. The number of cycles present during the diet treatment period was calculated.

### 2.3. Ovarian Histological Analysis

Excised ovaries were weighed and stored in 10% neutral buffer formalin solution. They were first dehydrated in increasing concentrations of ethanol (25%, 50%, 75%, 99.5% and 100%) and then embedded in paraffin. Paraffin blocks were then sectioned and trimmed using a Rotary Microtome RM225 by Leica Biosystems, and 5 μm thick section series were prepared on microscopic slides. These sections were deparaffinized using successively decreasing concentrations of ethanol (100%, 99.5%, 80% and 70%). After deparaffinization, sections were stained with hematoxylin–eosin (Hematoxylin QS, Vector Laboratories, Burlingame, CA, USA) and observed under an inverted microscope after air drying. Sample IDs of the photomicrographs of the ovarian sections were blinded and the numbers of cystic follicles and corpora lutea were counted.

### 2.4. Sex Hormone Concentration Analyses

Luteinizing hormone (LH), follicle stimulating hormone (FSH), estradiol and testosterone hormone concentrations were measured using enzyme-linked immunosorbent assay (ELISA) kits. For each kit, the protocols given by the vendors were followed. (LH: Cusabio, Houston, TX, USA; detection range 0.3 mLU/mL–60 mLU/mL, FSH: LifeSpan BioSciences Inc., Seattle, WA, USA; detection range 2.47–200 ng/mL, estradiol: BioVendor, Czech Republic; detection range 2.5–1280 pg/mL, testosterone: BioVendor, Czech Republic; detection range 0.1–25 ng/mL). Absorbance results were analyzed using MyAssays online software.

### 2.5. Soy Isoflavone Metabolite Concentration Analysis

The plasma daidzein, genistein and equol concentrations were analyzed using high-performance liquid chromatography (HPLC) as detailed previously with minute modifications [63]. Frozen plasma samples from rats were thawed, and 100 μL aliquots were mixed with 100 μL of hydrolysis buffer (0.1 mol/L sodium acetate, pH 5) with 0.1% (wt/vol) ascorbic acid and 0.01% (wt/vol) ethylenediaminetetraacetic acid (EDTA), 8 μL of glucuronidase and 4 μL of sulfatase. The reaction mixture was allowed to hydrolyze to glucuronide and sulfate metabolites at 37 °C for at least 15 h. Subsequently, 10 μL of an internal standard (formononetin, 5 μg/mL in dimethylsulfoxide), 120 μL of water, 75 μL of ammonium acetate buffer (1 mol/L, pH 7) and 83 μL of triethylammonium sulfate buffer (3 mol/L, pH 7) were added, and the samples were then heated to 60 °C for 10 min to facilitate the dissociation of isoflavones from plasma proteins; the mixture was then centrifuged. The deproteinized samples were passed over 0.5 g Sep-Pak C-18 cartridges (Nihon Waters, Tokyo, Japan) that had been previously washed with 10 mL of chloroform, 10 mL of methanol and 20 mL of water. The cartridges were washed with 5 mL of ammonium acetate buffer (10 mmol/L, pH 5) and 5 mL of water at room temperature. The absorbed isoflavones were eluted with 1.5 mL of methanol. The methanol effluent was evaporated to dryness under a gentle stream of nitrogen at 45 °C, dissolved in 100 μL of (40:60 vol/vol) methanol/aqueous acetic acid (1%), and stored at −20 °C until HPLC analysis.

The internal standard method of HPLC was used. After mixing with 5 mg/L formononetin, three concentration series of 0, 1, and 2 mg/L daidzein, genistein and equol were made. This was used to create the standard curve. A 5 μL aliquot of the sample was applied to a reversed-phase HPLC column (2.0 × 150 mm, particle size 5 μm from Unison US-C18, Imtakt, Portland, OR, USA). The mobile phase was used for all groups except for the PSR group, with potassium phosphate buffer containing 40% of a mixture of methanol and acetonitrile (3:2 vol/vol) at 40 °C. For the PSR group, potassium phosphate buffer containing 30% of a mixture of methanol and acetonitrile (3:2 vol/vol) at 40 °C was used. The flow rate used was 0.2 mL/min. Daidzein, genistein and equol were detected using an electrochemical detector (electrochemical detector 3005, Shiseido, Tokyo, Japan) under the following conditions: working electrode, glassy carbon; applied voltage, 800 mV. Formononetin was detected simultaneously using a UV–vis detector (SPD-10AV, Shimadzu Corporation, Kyoto, Japan) at 254 nm.

### 2.6. Gut Microbiota Analysis Using Next Generation Sequencing (NGS)

#### 2.6.1. Extraction of Bacterial Genomic DNA

The protocol followed was previously described in Inoue et al., 2017 [64]. Briefly, 25 mg of fresh cecal samples were collected and stored at −80 °C at the end of the study. Whole bacterial DNA was extracted from cecal contents using the QuickGene DNA Tissue kit SII (KURABO, Osaka, Japan), which is a DNA extraction kit for use with a semiautomated nucleic acid extraction machine (QuickGene810; KURABO).

#### 2.6.2. Library Preparation and DNA Sequencing

Library prepared as previously explained in Inoue et al., 2016 [64], and deep sequencing were performed using a MiSeq apparatus (Illumina K.K., Tokyo, Japan). Specifically, 341F and 805R primers with 5′ overhang adapter sequences for the second PCR (Polymerase Chain Reaction) were used to amplify the V3–4 region of 16S rRNA genes in each sample. NucleoFast 96 PCR plates (TaKaRa bio, Shiga, Japan) were used to purify the amplicons, and a unique combination of dual indices (I5 and I7 indices) was attached in the second round of PCR. After purification using a SequelPrep Normalization Plate Kit (Thermo Fisher, Tokyo, Japan), the concentration of each sample was normalized. Next, the samples were pooled and concentrated using AMPure XP beads (Beckman Coulter, Tokyo, Japan). Through the SequelPrep Normalization Plate Kit (Thermo Fisher, Tokyo, Japan), ten pM of the library combined with 20% phiX Control (Illumina) was sequenced with 285 bp paired-end bases on MiSeq.

#### 2.6.3. Sequence Data Analysis

Data of the sequences were processed using Quantitative Insights Into Microbial Ecology (QIIME) 2 (ver. 2020.8). Denoising was performed using the DADA2 plugin with the trimming length from the left set at 17 and from the right at 19. Truncation length was set to 250 for both reads. Taxonomic assignment was conducted through the Sklearn classifier algorithm against the Greengenes database 13_8 (99% Operational Taxonomic Units (OTUs) full-length sequences; available from https://docs.qiime2.org/, accessed on 22 October 2021). In this study, singletons and ASVs assigned to mitochondria and chloroplasts were removed by the command “feature-table filter-features” and “taxa filter-table” of QIIME2. The phylogenetic tree was generated via SATé-enabled phylogenetic placement (SEPP) [65]. The calculations of metrics regarding alpha and beta diversities were conducted by the command “diversity core-metrics-phylogenetic” of QIIME2 by setting the sampling depth at 5000 reads. The Chao1 index was calculated separately by the command “diversity alpha” of QIIME2 as the command “diversity core-metrics-phylogenetic” does not calculate this index.

Predictions of functional profiles from 16S rRNA genes were made using Phylogenetics Investigation of Communities by Reconstruction of Unobserved States (PICRUSt2) software and the Kyoto Encyclopedia of Genes and Genome (KEGG) database release 70.0 [66]. To obtain the data for processing picrust2 analyses, the following two files were exported from the QZA files that resulted in the above-mentioned QIIME2 analyses: the fasta formatted sequence list for representative sequences of ASVs and a biom file reflecting data of the ASV table. The former was simply achieved by the command “tools export”. For the latter, QZA files containing an ASV table and taxonomy of each ASV were first exported by the “tools export” command of QIIMEs and combined by a biom-format package of QIIME2. The entire pipeline was run with the “picrust2_pipeline.py” command and the inference of KEGG pathway abundances and the addition of a pathway name to KO IDs were made with “pathway_pipeline.py” and “add_descriptions.py” of the picrust2 package.

Default parameters were used for both QIIME2 and PICRUSt2 analyses unless otherwise stated.

### 2.7. Quantitative Reverse Transcription-PCR (qRT-PCR) Analysis of Gut Barrier Markers

#### 2.7.1. RNA Extraction

Total RNA was extracted from colon tissues. First, the tissues were homogenized in TRIzol reagent (Invitrogen, Tokyo, Japan), and total RNA was extracted using a Maxwell16 LEV simplyRNA kit (Promega, Tokyo, Japan).

#### 2.7.2. Complementary DNA (cDNA) Preparation through Reverse Transcription

Total RNA (0.5 µg) was reverse transcribed into cDNA using SuperScript IV VILO Master Mix (Thermo Fisher Scientific, Tokyo, Japan) according to the manufacturer’s recommendations. The synthesis of cDNA was performed in a total volume of 10 μL for 10 min at 25 °C and for 10 min at 50 °C. Then, the reaction was terminated by incubation at 85 °C for 5 min. Finally, a 10-fold dilution of cDNA was prepared by adding 90 µL of EASY Dilution (Takara Bio Inc., Tokyo, Japan) to the obtained solution.

#### 2.7.3. Real-Time Quantitative PCR (qPCR)

The 10-fold diluted cDNA solution was used as the template DNA. A Mic qPCR cycler by Biomolecular Systems, Queensland, Australia, was used for the analysis. In a Mic Tube (Biomolecular Systems, Queensland, Australia), 2 µL of template cDNA and 8 µL of reagents (PowerUpTM SYBR^TM^ Green Master Mix (Thermo Fisher Scientific, Tokyo, Japan): 5 µL, 5 µM Primer (Forward, Reverse): 1 µL, diethylpyrocarbonate (DEPC) water: 2 µL) were mixed. The mouse tubes containing 10 µL of PCR mix were then loaded into the Mic qPCR cycler. Settings were made to activate uracil-DNA glycosylases (UDGs) at 50 °C for 2 min and to activate dual-lock DNA polymerase at 95 °C for 2 min. After that, heat denaturation was carried out at 95 °C for 15 s, and annealing and elongation reactions were carried out at 60 °C for 1 min. The heat denaturation, annealing and elongation reactions were carried out for 45 cycles. All the primers used were predesigned and purchased from Takara Bio Inc., Tokyo, Japan (Table 2). The dissociation curve confirmed that only the target gene was amplified. The expression level of each gene was quantified based on the calibration curve prepared using the expression series of template cDNA. Each reaction was carried out in triplicate, and the average concentration values were calculated. Glyceraldehyde 3-phosphate dehydrogenase (GAPDH) was used as a loading control to normalize each sample.

### 2.8. SCFA Concentration Analysis

The measurement of SCFAs was performed using HPLC (LC-10AD; Shimadzu, Kyoto, Japan) by the internal standard method as described previously [67]. Approximately 300 mg of the cecal content was homogenized by vortex mixing in 2 mL of 10 mmol sodium hydroxide/L aqueous solution containing 2.5 mmol crotonic acid/L (Nakarai, Kyoto, Japan) as an internal standard in an ice-water bath and then centrifuged at 16,800× *g* at 4 °C for 15 min. The fat-soluble substances in the supernatant were removed by extraction with chloroform. The aqueous phase was filtered through a membrane filter (cellulose acetate, pore size 0.45 μm, DISMIC-13cp, Advantec Toyo Roshi, Tokyo, Japan). These samples were subjected to HPLC for the analysis of SCFAs. SCFAs were separated with an ion exclusion column and detected according to the postcolumn pH-buffered electroconductivity detection method [68] using an H-type cation exchanger column (shim-pack SCR-102H, 8 mm i.d. × 30 cm long; Shimadzu, Kyoto, Japan), column temperature 40 °C, with a mobile phase of 5 mmol p-toluene sulfonic acid/L aqueous solution (flow rate: 0.8 mL/min). Each SCFA separated by the column was mixed with a pH-buffer solution of 20 mmol Bis-Tris/L aqueous solution containing 5 mmol p-toluene sulfonic acid/L and 100 μmol EDTA/L (flow rate 0.8 mL/min, 40 °C) and was then detected using an electroconductivity detector of positive polarity at 40 °C (CDD-6A; Shimadzu).

### 2.9. Statistical Analysis

Significant differences and normality of the physiological features were analyzed using IBM’s statistical package for the social sciences (SPSS) statistics v20.0 software. One-way analysis of variance (ANOVA) followed by the correction of *p* values with Dunnett’s test was used to determine significant differences between those data. Differences in the abundance of bacterial genera and Kyoto Encyclopedia of Genes and Genomes (KEGG) pathways between groups were analyzed by Kruskal–Wallis non-parametric test and the significant differences were measured by the Tukey–Kramer test using Software Testing Amplification (STAMP) software [69]. Correlations of the bacterial abundance with PCOS-like reproductive features were examined by Spearman’s non-parametric test using JMP Pro v13.0 software. All data were expressed as the mean ± SEM. *p* < 0.05 was considered statistically significant, and *p* < 0.10 was considered to indicate a tendency.

## 3. Results

### 3.1. Analysis of the Three Main PCOS Diagnostic Features

**Figure 2:**
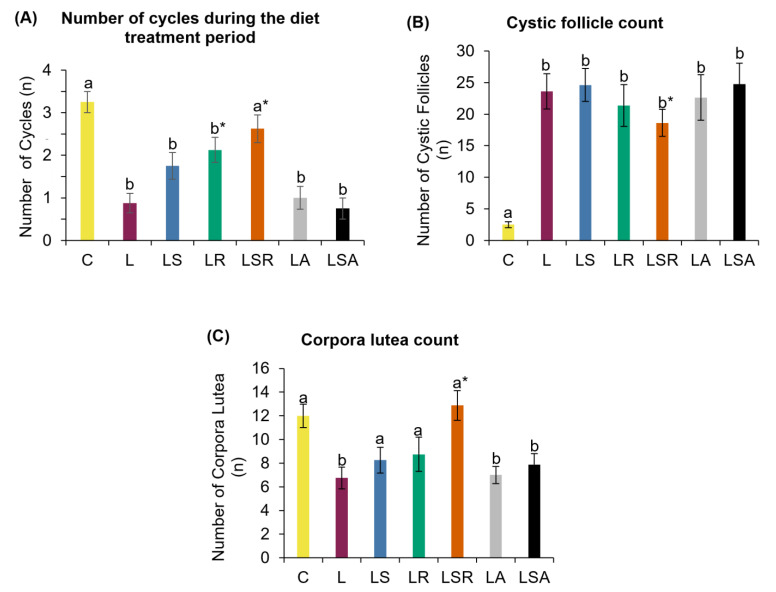
Analyses of menstrual irregularity and polycystic ovaries. (**A**) Number of cycles present during the diet treatment period. This analysis was performed to measure menstrual irregularity. Vaginal cell suspensions were collected daily and stained using Modified Wright–Giemsa stain to identify the cycle stage on each day. The number of cycles was counted during the 21-day diet treatment period. (**B**) Cystic follicle count and (**C**) corpora lutea count were measured using ovarian histological analysis. Excised ovaries were embedded in paraffin blocks, and the sections were stained using hematoxylin–eosin stain. All the values are expressed as the mean ± S.E. Statistical analysis was conducted by one-way ANOVA followed by Dunnett’s test for multiple comparison analysis. a, b represent *p* < 0.05 compared to the C group, * represents *p* < 0.05 compared to the L group.

We first analyzed the three main PCOS diagnostic features. Menstrual cycles were examined via histochemical analysis of daily vaginal samples. We observed a normal 4–5-day menstrual cycle in the control group with distinctive cells in the four stages: proestrus, estrus, metestrus and diestrus (Supplementary Figure S1). As shown in Figure 2A, during the 3 weeks of the treatment period, all letrozole-treated groups except the LSR group displayed a significantly lower number of cycles than the C group. The LR and LSR groups displayed a significantly higher number of cycles compared to the L group. The LS group and the antibiotic-treated groups (LA and LSA) did not show any notable changes in the number of cycles compared to the L group. During the treatment period, we observed that L, LA and LSA groups started to show acyclicity within one week from the treatments. In contrast, LS, LR and LSR groups displayed prolonged cycles with an average of 6–8 days.

**Figure 3:**
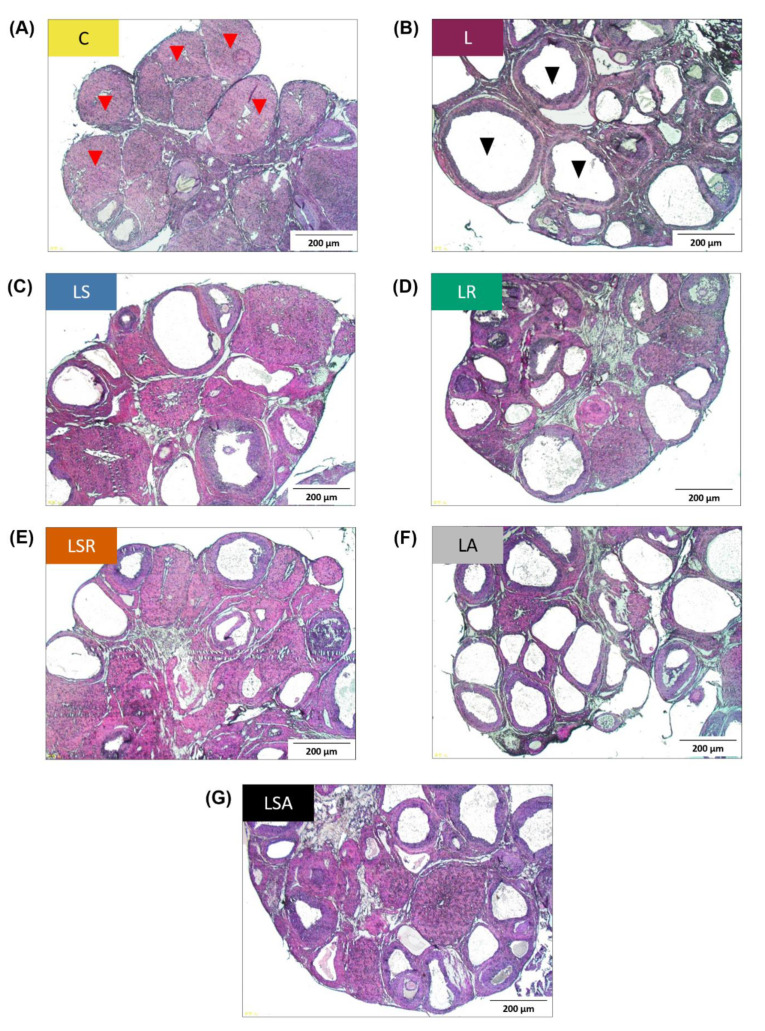
Photomicrographs of ovarian sections of a representative rat from each study group. Excised ovaries were embedded in paraffin blocks, and the sections were stained using hematoxylin–eosin stain. (**A**) C group, (**B**) L group, (**C**) LS group, (**D**) LR group, (**E**) LSR group, (**F**) LA group, (**G**) LSA group. Red arrowheads = corpora lutea; black arrowheads = cystic follicles; scale bars = 200 μm.

Excised ovaries were embedded in paraffin, sectioned and stained. Different types of follicles were counted in the stained sections to assess the status of polycystic ovaries. Cystic follicle and corpora lutea counts revealed that the number of cystic follicles was significantly higher in the letrozole-treated groups than in the C group. The number of corpora lutea was significantly lower in the L group and the antibiotic-treated groups (LA, LSA) than in the C group. In contrast to the L group, a significant reduction in the onset of cyst formation in the LSR group was detected. The number of corpora lutea observed in the LSR group was also significantly higher than that of the L group (Figure 2B,C). Figure 3 shows photomicrographs of ovarian sections of one representative rat from each group.

**Figure 4:**
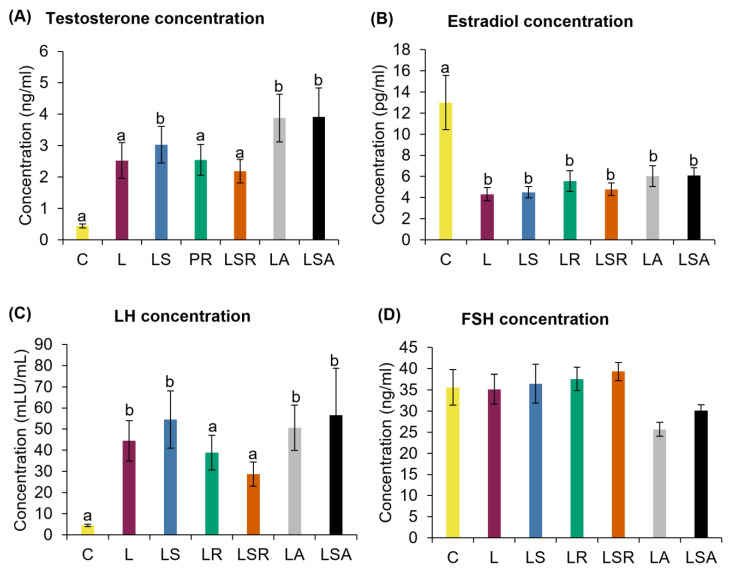
Analyses of reproductive hormones. Biochemical analyses of reproductive hormones; (**A**) testosterone, (**B**) estradiol and pituitary hormones; (**C**) LH and (**D**) FSH. These hormones were measured in the serum samples of the rats using the respective ELISA kits. All the values are expressed as the mean ± S.E. Statistical analysis was conducted by one-way ANOVA followed by Dunnett’s test for multiple comparison analysis. a, b represent *p* < 0.05 compared to the C group.

Hyperandrogenism was measured by reproductive (testosterone, estradiol) and pituitary (LH and FSH) hormonal concentration analyses using the respective ELISA kits. LS, LA and LSA groups displayed a significantly high testosterone concentration compared to the C group. However, no discernible changes were observed compared to the L group. Estradiol concentration analysis revealed that all study groups had a significantly lower concentration than the C group. Similar to testosterone, we did not see any notable differences in estradiol concentrations in the study groups compared to the L group. (Figure 4A,B). In contrast to the C group, the LH concentration was significantly higher in the L group. FSH concentration analysis did not reveal any notable trends (Figure 4C,D).

### 3.2. Daidzein and Equol Concentration Analyses

**Figure 5:**
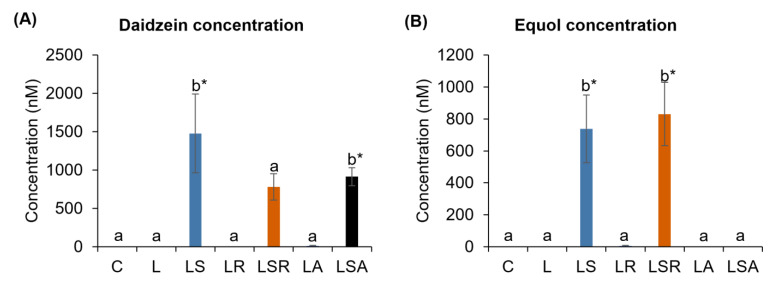
Daidzein and equol concentration analyses. Soy isoflavone metabolite concentrations in plasma were analyzed via HPLC. (**A**,**B**) show daidzein and equol concentrations, respectively. All the values are expressed as the mean ± S.E. Statistical analysis was conducted by one-way ANOVA followed by Dunnett’s test for multiple comparison analysis. a, b represent *p* < 0.05 compared to the C group, * represent *p* < 0.05 compared to the L group.

Concentrations of the soy isoflavone metabolites were analyzed through HPLC methodology to understand their effects on the PCOS-like symptoms. Our results revealed that the equol level was high in the LSR group compared to the LS group (but not significantly), and equol was not detected in the LSA group. A high level of daidzein was detected in the LS group compared to those levels in the LSR and LSA groups (Figure 5).

### 3.3. Analysis of Gut Microbiota Profiles

**Figure 6:**
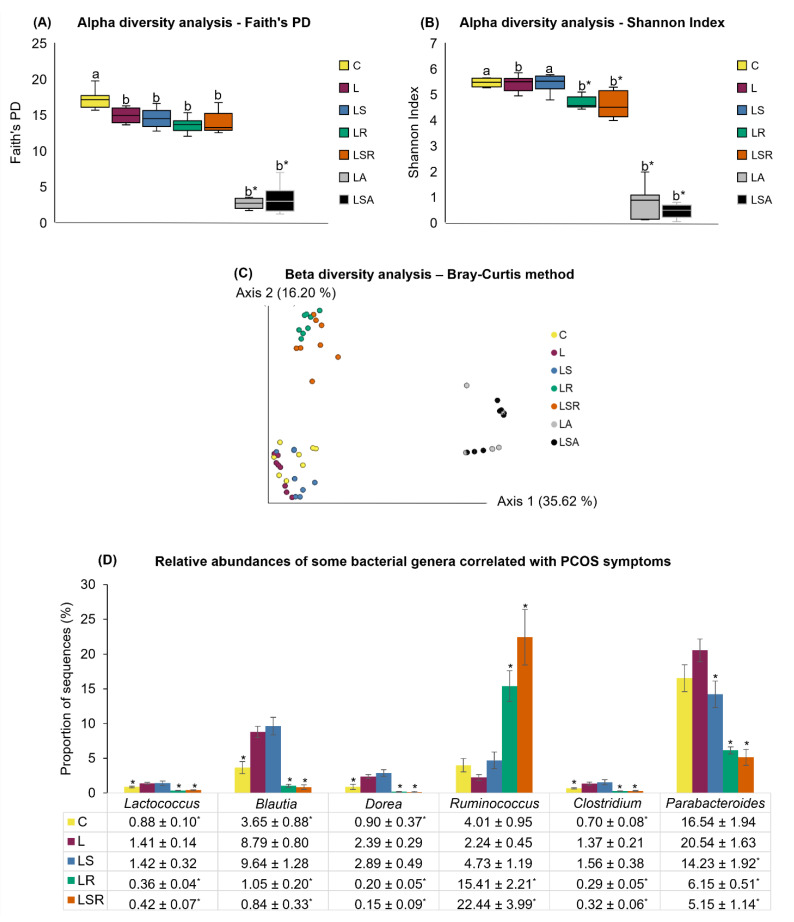
Analyses of gut microbial profiles. The cecal content of each rat, which was collected at the end of the experiment, was used to analyze the gut microbiota composition using 16S rRNA sequencing. Alpha diversity analysis by (**A**) Faith’s PD index and (**B**) Shannon index. (**C**) Beta diversity analysis by Bray–Curtis method. (**D**) Relative abundances of significantly different bacterial genera. All the values are expressed as the mean ± S.E. Statistical analysis was conducted by Kruskal–Wallis test followed by Tukey–Kramer test for multiple comparison analysis. a, b represent *p* < 0.05 compared to the C group, * represent *p* < 0.05 compared to the L group.

We also performed a thorough analysis of gut microbiota profiles to investigate their possible relationships with PCOS symptoms. Next-generation sequencing and statistical tools such as STAMP and JMP were used in this analysis. Figure 6A shows the results of alpha diversity analysis using Faith’s Phylogenetic Diversity index. According to the plot, the alpha diversity was significantly decreased in the L group compared to the C group. Similarly, the LR, LSR, LA and LSA groups also displayed a significantly lower alpha diversity than the C group, while only the LA and LSA groups displayed a significantly lower alpha diversity than the L group. Evenness was analyzed using Shannon index (Figure 6B). The results revealed that except for the LS group, all other groups showed a significantly low alpha diversity compared to the C group. LR, LSR, LA and LSA groups showed a significantly low alpha diversity compared to the L group. Further analysis of alpha diversity was carried out using the Chao1 index (Supplementary Figure S2). Weighted and unweighted beta diversity was measured in the C and L groups. A significant difference was only observed in the unweighted beta diversity measurement (Supplementary Figure S3). Bray–Curtis analysis displayed a notable difference in the beta diversity of antibiotic-treated groups (LA, LSA) and resistant starch-treated groups (LR, LSR) compared with the other three groups: C, L and LS (Figure 6C).

The genus-level differences between the C and L groups showed that bacterial genera such as *Blautia*, *Dorea*, *Lactococcus* and the genus *Clostridium* of the family Erysipelotrichaceae were significantly more abundant in the L group than in the C group (Figure 6D).

Some bacterial genera such as *Blautia*, *Dorea*, *Lactococcus*, *Allobaculum*, and the genus *Clostridium* of the family Erysipelotrichaceae were negatively correlated, and Coprococcus and the genus *Ruminococcus* of the family Ruminococcaceae were positively correlated with the number of cycles during the diet treatment period. Genera *Blautia*, *Dorea*, *Lactococcus* and genus *Clostridium* of the family Erysipelotrichaceae were positively correlated, and the genus *Ruminococcus* of the family Ruminococcaceae was negatively correlated with the number of cysts. Although the testosterone concentrations of C and L groups were not significantly different with each other, correlation analysis revealed that genera *Blautia*, *Lactococcus* and genus *Clostridium* of family Erysipelotrichaceae were positively correlated with testosterone concentration (Table 3).

Genus-level relative abundance analyses of the LR and LSR groups versus the L group showed that the genera *Blautia, Dorea*, *Lactococcus*, *Parabacteroides* and genus *Clostridium* of the family Erysipelotrichaceae had a significantly lower relative abundance in the LR and LSR groups than in the L group. Additionally, the relative abundance of the genus *Ruminococcus* was significantly higher in the LR and LSR groups than in the L group. Moreover, when comparing the significantly different relative abundances of bacterial genera in the LS and L groups, the genus *Parabacteroides* was significantly lower in the LS group than in the L group. The comparative analysis of the relative abundances of bacterial genera in the LS and LSR groups revealed that the genera *Blautia, Dorea*, *Lactococcus*, *Parabacteroides* and genus *Clostridium* of the family Erysipelotrichaceae were significantly less abundant and that the genus *Ruminococcus* was significantly more abundant in the LSR group than in the LS group. A summary of the relative abundances of the bacterial genera explained above is shown in Figure 6D.

### 3.4. Analysis of Gut Barrier Markers

**Figure 7:**
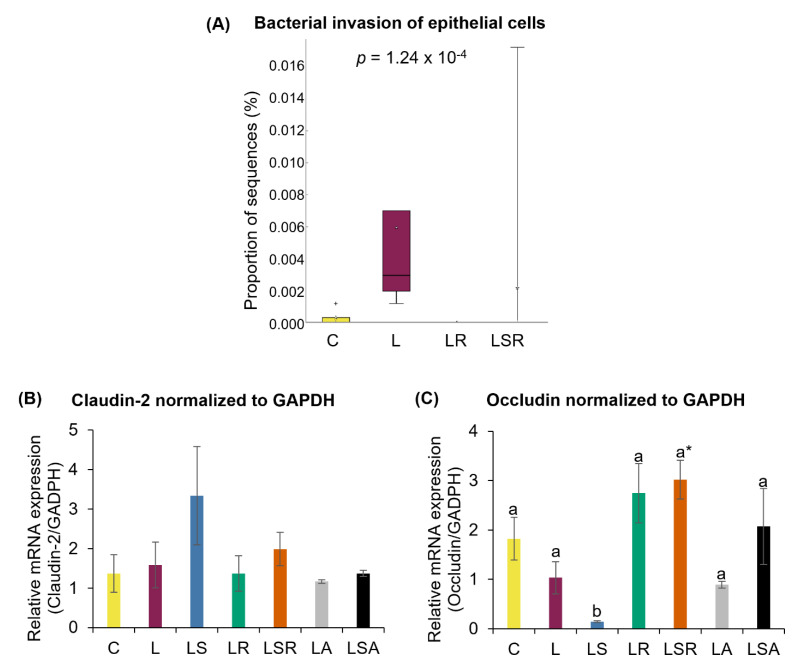
Analyses of gut barrier markers. (**A**) Relative abundance of microbial functional pathway responsible for bacterial invasion of epithelial cells. This analysis was performed by evaluating the differences in the function of the microbial communities ascertained from 16S rRNA sequencing using PICRUSt software. Relative mRNA expression of gut barrier markers; (**B**) claudin-2 and (**C**) occludin. The expression levels of the genes in the colon samples were measured using quantitative RT-PCR. GAPDH was used as a loading control to normalize each sample. All the values are expressed as the mean ± S.E. Statistical analysis of bacterial sequences was conducted by Kruskal–Wallis test followed by Tukey–Kramer test for multiple comparison analysis. Statistical analysis of other parameters was conducted by one-way ANOVA followed by Dunnett’s test for multiple comparison analysis. a, b represent *p* < 0.05 compared to the C group, * represent *p* < 0.05 compared to the L group.

Relative abundances of microbial functional pathways were analyzed to determine the effects of gut microbiota on various mechanisms. This was performed by evaluating the differences in the function of the microbial communities ascertained from 16S rRNA sequencing using PICRUSt software. There were significant differences between the microbial functional pathways of the C and L groups. The proportion of genes responsible for bacterial invasion into intestinal epithelial cells was significantly increased in the L group, suggesting leaky gut status. The proportions of genes responsible for bacterial invasion into intestinal epithelial cells in the LR and LSR groups were significantly reduced compared to those in the L group (Figure 7A and Supplementary Figure S4). Levels of claudin-2 and occludin did not change significantly between the C and L groups. However, interestingly, the relative mRNA expression level of occludin in the LSR group was significantly higher than that in both the C and L groups (Figure 7B,C).

### 3.5. Analysis of SCFAs

**Figure 8:**
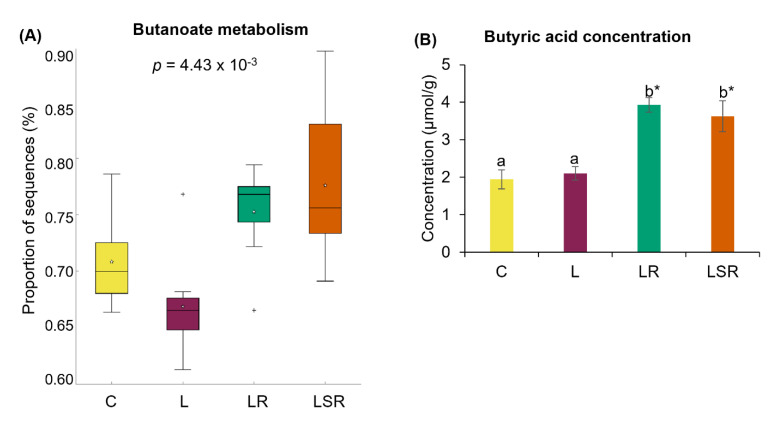
Analyses of SCFAs. (**A**) Relative abundance of microbial functional pathway responsible for butanoate metabolism. This analysis was performed by evaluating the differences in the function of the microbial communities ascertained from 16S rRNA sequencing using PICRUSt software. (**B**) Butyric acid concentration. Concentrations of the SCFAs were measured using the cecal content samples. Supplementary Figure S5 shows the concentrations of acetic acid and propionic acid. All the values are expressed as the mean ± S.E. Statistical analysis was conducted by one-way ANOVA followed by Dunnett’s test for multiple comparison analysis. Statistical analysis of bacterial sequences was conducted by Kruskal–Wallis test followed by Tukey–Kramer test for multiple comparison analysis. Statistical analysis of other parameters was conducted by one-way ANOVA followed by Dunnett’s test for multiple comparison analysis. a, b represent *p* < 0.05 compared to the C group, * represent *p* < 0.05 compared to the L group.

In comparison to the L group, the proportion of microbial genes responsible for butanoate metabolism was significantly high in both the resistant starch-treated LR and LSR groups (Figure 8A). To further examine the effects of SCFAs, we measured the concentrations of acetic acid, propionic acid and butyric acid using HPLC. We found that butyric acid concentrations in the LR and LSR groups were significantly higher than those in the C and L groups (Figure 8B). However, acetic and propionic acid concentrations did not show any notable changes among the groups (Supplementary Figure S5).

### 3.6. Analysis of Metabolic Syndrome Parameters

As obesity is a main phenotype in the PCOS population, we analyzed the body weights, reproductive organ weights and serum metabolic parameters (alanine aminotransferase, aspartate transaminase, total cholesterol, triglycerides, non-esterified fatty acids, low-density lipoprotein (LDL) cholesterol, high-density lipoprotein (HDL) cholesterol and total ketone bodies). This analysis was carried out by Oriental Yeast Co. Ltd., Tokyo, Japan. Body weight analysis showed that at the end of the letrozole treatment period, the average body weights in the L, LS and LR groups were significantly higher than those in the C group (Supplementary Table S2). There were no discernible changes observed in the ovarian weights among the groups (Supplementary Figure S6A). However, the C group displayed a significantly higher uterine weight than all other groups, suggesting uterine hypertrophy (Supplementary Figure S6B). The total cholesterol concentration was significantly reduced in both the resistant starch-treated groups (LR and LSR) and the LSA group compared to the C group. Interestingly, the PR group displayed a significantly lower total cholesterol concentration than the L group. Other metabolic parameters did not show any considerable changes (Supplementary Figure S7).

## 4. Discussion

Most intervention studies that have used a letrozole-induced PCOS animal model have carried out the interventions after the letrozole treatment period [29]. Recent evidence has shown that once the letrozole treatment was stopped, the PCOS-like features were reversed [70]. To overcome this concern in our study, we analyzed the effects of soy isoflavones, resistant starch and antibiotics on the severity of PCOS-like features while simultaneously treating rats with letrozole. Moreover, compared to previous studies that examined the effects of soy isoflavones on PCOS, we examined the effects of soy isoflavones and equol both by altering soy isoflavone metabolism through the addition of resistant starch and antibiotics. In addition, we could also evaluate the individual effects of resistant starch and antibiotics, which have not been well studied to date.

Our results revealed that letrozole treatment was successful in inducing two out of the three main PCOS diagnostic criteria: menstrual irregularity and polycystic ovaries. After treatment with resistant starch alone and in combination with soy isoflavones, we observed a notable suppression in the severity of menstrual irregularity. In an animal study performed by Rajan et al. (2017) [29], it was reported that soy isoflavones can reduce the percentage of diestrus days, which is an indicator of menstrual irregularity. In contrast, Patisaul et al. (2014) [35] reported that a soy-based diet given throughout the gestational and postnatal periods can induce menstrual irregularity in rats. Furthermore, a PCOS clinical study that used 36 mg/day genistein for 6 months reported that genistein treatment did not change menstrual irregularity significantly [34].

We observed a larger number of cystic follicles and a smaller number of corpora lutea, which could be seen in letrozole-induced rats, as reported earlier [5,10,33]. Kafali et al. (2004) [33] suggested that these histologic changes are due to the presence of biologically active levels of FSH, increased LH and a lack of interplay between granulosa cells. According to Rajan et al. (2017) [10], soy isoflavone (100 mg/kg) treatment showed protective effects in ovarian histological sections. In the present study, we observed that the development of the number of cystic follicles tended to decrease in the soy isoflavone-treated group (*p* = 0.085) and the resistant starch-only treated group (*p* = 0.059). Interestingly, both the soy isoflavone- and resistant starch-treated groups displayed a significant reduction in the number of cystic follicles and a significant increase in the number of corpora lutea compared to the letrozole-treated PCOS group (L).

Through steroid hormone analyses, we observed that the L group displayed an elevated tendency toward testosterone concentration (*p* = 0.076) and a significant reduction in the estradiol concentration. However, after the diet treatments, we could not observe any significant changes in the steroid hormone concentrations. In contrast to our results, some animal and clinical studies reported that soy isoflavone administration can reduce hyperandrogenism through reduced testosterone levels [29,30,31,71]. However, Romualdi et al. (2008) [34] reported an increasing trend in androstenedione and testosterone levels in PCOS patients who consumed genistein. In our study, although we observed a significantly high LH level in the L group, the FSH concentration did not vary in a notable manner among the groups. Khani et al. (2011) [31] reported that genistein administration did not significantly change FSH levels in PCOS patients. Haudum et al. (2020) [32] demonstrated a lower LH:FSH ratio in PCOS patients with higher baseline genistein levels.

According to the soy isoflavones and their metabolite concentration analyses, the group treated with soy isoflavone only (LS) had a high concentration of daidzein and a low concentration of equol compared to the group treated with both soy isoflavones and resistant starch (LSR). These results suggest that resistant starch was able to increase soy isoflavone metabolism. Resistant starch is believed to enhance the metabolism of soy isoflavone conjugates to their aglycone forms and the metabolism of aglycone:daidzein to equol by stimulating the activity of β-glucosidase [56]. Moreover, a high concentration of genistein and a low concentration of daidzein without any presence of equol in the soy isoflavone- and antibiotic-treated group (LSA) were also observed. This implies that antibiotic treatment is able to diminish the gut microbiota responsible for metabolizing soy isoflavones.

Looking at the above-discussed results related to reproductive PCOS parameters, it can be understood that soy isoflavones in combination with resistant starch showed the most positive effects on lowering the severity of PCOS-like features. It is clear that with increasing equol concentrations, the development of PCOS-like features is controlled better. Lacey et al. (2005) [72] suggested that the inhibition of the expression and activity of 17β-HSD by phytoestrogens may result in decreased levels of testosterone. Another study suggested that the reduction in the percentage of diestrus days in soy-treated PCOS rats might be due to their ability to decrease testosterone concentrations in the peripheral blood [29]. Most of these suggested mechanisms are related to the link between soy isoflavones and equol and hyperandrogenism. However, in our study, we observed more hyperandrogenism in the soy isoflavone-treated group (LS) compared to the PCOS group (L). It is difficult to make a direct comparison between clinical and animal studies, and due to the differences between animal studies themselves (species, age, diet compositions, duration of the diet treatments, etc.), drawing conclusions is difficult.

A recent study that used resistant dextrin as a diet treatment in PCOS patients revealed that resistant dextrin consumption can alleviate hyperandrogenism and menstrual irregularity in PCOS women [73]. These results are in line with our study, which showed that resistant starch treatment was successful in lowering the severity of menstrual irregularity.

Our second hypothesis was that bacterial invasion into gut mucous caused by increased gut permeability or leaky gut is an important effector of PCOS pathogenesis. The NGS results revealed that there is a broad view of the association between PCOS and gut microbiota in PCOS rats. We noted a significant reduction in alpha diversity and a distinctive difference of beta diversity in our letrozole-treated PCOS group (L). In accordance with our results, Kelley et al. (2016) [3] suggested that due to the effect of letrozole, Faith’s phylogenetic diversity was reduced in PCOS mice. Additionally, some other human studies showed that the species and phylogenetic diversity decreased in PCOS patients compared to that in healthy patients [4,39]. While a significant reduction in the alpha diversity of antibiotic-treated groups (LA and LSA) was clear, a reduced alpha diversity in the groups treated with the soy isoflavone and resistant starch mix (LR = significant reduction and LSR) was also observed. They also showed distinctive clustering in β-diversity analysis. Hu et al. (2016) [74] reported that when resistant starch was fed to rats with colitis-associated colorectal cancer, the bacterial diversity was reduced at the phylum and species levels (measured through the Shannon index).

Some discernable differences in the bacterial genera belonging to the L group compared to those of the control group and in the LS, LR and LSR groups compared to the PCOS group were observed in our study. According to the results of Guo et al. (2016) [45], *Lactobacillus*, *Ruminococcus* and *Clostridium* levels were lower and *Prevotella* levels were higher in the PCOS group. Furthermore, a few other studies reported that bacterial genera such as *Roseburia*, *Dorea*, *Allobaculum*, *Coprobacillus* and *Blautia* were significantly increased and genera such as *Akkermansia*, *Lactobacillus*, *Parabacteroides* and *Alistipes* were significantly decreased in PCOS animals [3,46,70]. Some clinical studies also reported significant differences at the phylum and genus levels in PCOS patients compared to controls [4,39,40,41,75]. In our study, we noted a few trends similar to those reported previously, such as a high relative abundance of the genera *Blautia* and *Dorea* in the -L group. Due to the huge variability among these studies with respect to animal models, the duration of treatments, etc., it is difficult to make direct comparisons between these findings. However, it can be well understood that gut microbiota are significantly altered in PCOS patients and animals.

Interestingly, after the diet treatments, we found that resistant starch-treated groups showed opposite trends in some bacterial genera that were reported in the L group, such as *Blautia*, *Dorea*, *Lactococcus* and *Clostridium* of the family Erysipelotrichaceae. Moreover, the genera *Ruminococcus* and the family S24–7 were significantly abundant in the resistant starch-treated groups. *Ruminococcus* spp. have been previously shown to play an important role in resistant starch degradation [76,77]. Additionally, *Ruminococcus bromii* was reported to be associated with insulin sensitivity and increase concentrations of SCFAs [51,78]. Reports of gut microbiota analyses in PCOS studies with soy isoflavone treatments are limited. A very recent study reported that short-term isoflavone intervention in PCOS patients increased the concentrations of the genera *Oscillospira*, *Magasphaera*, *Parabacteroides* (*Parabacteroides disrasonis*), etc. In contrast, we noted that the genus *Parabacteroides* was significantly reduced in the group treated with soy isoflavone only (LS) in our study. Through the NGS method used in our study, precise bacterial species could not be identified. However, when comparing the results of LS, LR and LSR groups, we found that the bacterial genera which were significantly different (less abundant compared to the L group) in the LS group were also observed in both LR and LSR groups. These results imply that although they are metabolized by gut microbiota, soy isoflavones alone do not have a significant effect on the gut microbiota, which contributes to the reduction of the PCOS-like features. To get a clearer idea on the actual amount of gut microbiota that were changed, we suggest that the quantification of 16S rRNA gene should be carried out.

Correlation analyses performed in PCOS animal- and human-based studies have revealed beneficial information about the association between gut microbiota and metabolic and reproductive parameters of PCOS. Kelley et al. (2016) [3] reported a negative correlation between testosterone and alpha diversity, suggesting that hyperandrogenemia is responsible for decreasing the bacterial species abundance in the large intestine. In contrast, Torres et al. (2019) [46] revealed that α-diversity was not restored in letrozole-treated mice after cohousing them with placebo mice. A number of human-based studies have also reported significantly positive and negative correlations with bacteria, such as the phylum Tenericutes, order ML615J-28, S24–7 family, *Bacteroidaceae* family, *Prevotellaceae* family, *Coprococcus*, *Ruminococcus, and Faecalibacterium*, and hyperandrogenism [4,42,47,75]. In comparison with previous reports, our study results revealed that genera such as *Blautia*, *Dorea*, *Lactococcus* and genus *Clostridium* of the family Erysipelotrichaceae were negatively correlated and genera *Ruminococcus*, *Coprococcus* were positively correlated with menstrual cyclicity; the genera *Blautia*, *Dorea*, and genus *Clostridium* of the family Erysipelotrichaceae were positively correlated and the genus *Ruminococcus* was negatively correlated with cysts in the ovaries. The relative abundances of these bacteria in the resistant starch-treated groups and the decreased severity of PCOS symptoms in them supports the correlation between these bacteria and PCOS symptoms. 

Using metagenome statistics, we were able to establish the significant contribution of some PCOS-related microbial pathways to the severity of PCOS symptoms. The significantly high relative abundance of the genes associated with bacterial invasion into intestinal epithelial cells in the L group suggested potential leaky gut status. To further confirm this, we analyzed the relative mRNA expression levels of some gut barrier markers, such as claudin-2 and occludin, in colon samples. Reports on gut permeability related to PCOS are limited. Through the DOGMA theory, Tremellen and Pearce (2012) [38] postulated that dysfunction in gut barrier function leading to chronic inflammation is an important effector in inducing PCOS symptoms. There are contradictory reports on the link between gut barrier markers and PCOS. While Zhang et al. (2015) [79] and Lindheim et al. (2017) [4] reported that zonulin and LBP were increased in PCOS patients, Zeynep et al. (2019) [80] reported the opposite. In comparison to these reports, our L group did not show any significant changes in claudin-2 and occludin expression. After diet treatments, the only discernible change we observed was the increase in occludin expression in the LSR group compared to that in the PCOS group (L). This was further confirmed by the presence of a significantly low relative abundance of the genes related to bacterial invasion into the gut reported in the LSR group. Additionally, another observation was that soy isoflavones alone decreased the occludin concentration in contrast to an increased concentration when mixed with resistant starch. Luo et al. (2019) reported that a high soy isoflavone diet (450 mg/kg) can reduce the occludin concentration in high-fat-diet-induced obese rats [81]. There are previous reports stating the beneficial effects of resistant starch in preventing type 2 diabetes and obesity through improving gut barrier function [82]. While it is unclear why occludin concentration was reduced in our LS group, it can be understood that the effect of resistant starch and high equol levels in improving gut barrier function in the LSR group was considerably high.

Moreover, the increased abundance of the genes associated with butanoate metabolism in the resistant starch-treated groups (LR and LSR) confirms the ability of resistant starch to produce SCFAs. The high plasma butyric acid concentration in the LR and LSR groups also proved this. Of all the SCFAs, butyrate has been studied most extensively for its role in nourishing the colonic mucosa and maintaining gut barrier function [82]. A meta-analysis indicated that in PCOS patients, abnormal SCFA metabolism was caused by disrupted gut microbiota, which is linked to insulin resistance and hyperandrogenemia [83]. Zhao et al. (2020) [13] concluded that gut microbiota is linked to the development of PCOS symptoms through the SCFA pathway.

There are controversies surrounding the reports on antibiotic usage in animals. While some reports stated that antibiotic treatments can lower gut permeability and metabolic endotoxemia [84,85], others have reported that antibiotic treatments can induce mucosal permeability and mucin expression [86,87]. The opposite results reported in these studies could be due to the variations in the animal models, antibiotic dosages used and the length of the treatments.

In relation to obese phenotypes, we observed significantly high body weight and uterine hypotrophy in PCOS rats. The body weights of the soy isoflavone- and resistant starch-treated groups were decreased but not in a significant manner. However, total cholesterol levels in the group treated with resistant starch only (LR) were significantly reduced compared to those in the PCOS group. Supporting our results, Shamasbi et al. (2019) [73] reported that resistant dextrin reduced total cholesterol, LDL cholesterol and triglycerides and increased HDL cholesterol in PCOS patients.

Overall, through our study, we found that some gut microbiota were associated with PCOS features and that the leaky gut status was not supported by sufficient evidence. Our diet treatments revealed that higher equol concentrations had a direct effect on suppressing the severity of cyst formation to a certain extent. The effects of soy isoflavone and equol on modulating the gut microbiota linked to PCOS were minute. On the other hand, resistant starch showed promising results in lowering the severity of PCOS features by modulating the gut microbiota linked to PCOS. Additionally, resistant starch modulated the gut microbiota, which affected two pathways: SCFA production and bacterial invasion into gut epithelia. These pathways have been linked to PCOS pathogenesis in previous reports [13]. Antibiotic treatments used in our study did not show any considerable changes in the PCOS features. In a recent study carried out by Yang et al. (2021), mice were treated with letrozole and an antibiotic cocktail for 5 weeks [88]. The antibiotic cocktail contained the same antibiotics but in higher concentrations (20 mg/mL vancomycin, 40 mg/mL neomycin sulfate, 40 mg/mL metronidazole, and 40 mg/mL ampicillin) compared to the antibiotic cocktail that was used in our study. They reported that the letrozole- and antibiotic-treated mice showed a significantly lower testosterone concentration [88]. These results are contradictory with our results and one possible reason for that could be the lower concentrations and the shorter duration of the antibiotic treatment used was not sufficient to make a significant effect in lowering the testosterone concentration. We suggest that the dosages and the duration of the antibiotic treatments are significantly important in investigating their effects on PCOS symptoms. 

As the most common endocrine disorder in women with a diverse etiology, there is a timely need to find potential therapies for PCOS. Functional foods have attracted growing interest worldwide as alternative therapies for diseases. Hence, our study could be of great interest to researchers, health professionals, PCOS patients and even to other people who are concerned about PCOS. As with any animal model, PCOS animal models also have their pros and cons. We selected the letrozole-induced animal model after an intensive literature review [89,90,91]. Our principal focus was on its ability to induce the main PCOS-like reproductive features, usage in intervention studies and the feasibility of the method. According to a review by Ryu et al. (2019) the aromatase inhibitor letrozole provides good reproducibility for PCOS-like features in rodents [92]. However, the letrozole-induced PCOS animal model does not fully manifest all the PCOS symptoms displayed in humans, especially the metabolic features, and the variability among the features in letrozole-induced PCOS studies is also high [92]. Considering these drawbacks, further clinical investigations are required to understand the effects and the exact mechanisms of action of our diet treatments in humans.

To the best of our knowledge, this is the first time the individual and combined effects of soy isoflavones, equol, resistant starch and antibiotics on PCOS symptoms were studied. In conclusion, we believe the novel dietary therapies suggested in this study will be beneficial as an alternative treatment method for PCOS.

## Figures and Tables

**Table 1:** Diet composition.

	Control		Iso		RS		Iso+RS	
	AIN-93G Powder		Isoflavone (0.05%)		Resistant Starch (11%)		ISO (0.05%), RS (11%)	
	**gm%**	**kcal%**	**gm%**	**kcal%**	**gm%**	**kcal%**	**gm%**	**kcal%**
Protein	20.3	20.3	20.3	20.3	20.3	20.3	20.3	20.3
Carbohydrate	63.9	63.9	63.9	63.9	63.9	63.9	63.9	63.9
Fat	7.00	15.8	7.00	15.8	7.00	15.8	7.00	15.8
Total		100.0		100.0		100.0		100.0
kcal/gm	4.00		4.00		4.00		4.00	
**Ingredient**	**gm**	**kcal**	**gm**	**kcal**	**gm**	**kcal**	**gm**	**kcal**
Casein	200	800	200	800	200	800	200	800
L-Cystine	3	12	3	12	3	12	3	12
Corn Starch	397.486	1589.9	396.486	1585.9	197.486	789.9	196.486	785.9
Maltodextrin	132	528	132	528	132	528	132	528
Sucrose	100	400	100	400	100	400	100	400
Cellulose, BW200	50	0	50	0	50	0	50	0
Corn Oil	70	630	70	630	70	630	70	630
t-Butylhydroquinone	0.014	0	0.014	0	0.014	0	0.014	0
Mineral Mix S10022G	35	0	35	0	35	0	35	0
Vitamin Mix V10037	10	40	10	40	10	40	10	40
Choline Bitartrate	2.5	0	2.5	0	2.5	0	2.5	0
Soyaflavone HG (Fuji Oil)			1	0			1	0
HI-MAIZE 260 (Ingredion)					200	800	200	800
**Total**	**1000**	**3999.9**	**1000**	**3995.9**	**1000**	**3999.9**	**1000**	**3995.9**

**Table 2:** List of forward and reverse primers used in real-time qPCR.

Primer	Primer ID	Primer Sequence
Occludin9-Forward	RA055910-F	GGTGCCATAGAATGAGATTGGA
Occludin-Reverse	RA055910-R	CCAATGGGCACACCCTGATAC
Claudin2-Forward	RA034042-F	ATTCGAGTCATCGCCCATCAG
Claudin2-Reverse	RA034042-R	CCAGGCAGAAGTTCACCAATCA
GADPH-Forward	RA015380-F	ATTCCTGGACCCAAAACGCT
GADPH-Reverse	RA015380-R	CCACCAACTGCTTAGCCCCC

**Table 3:** Some correlations between the relative abundance of bacteria and menstrual irregularity (number of cycles during the diet treatment period), polycystic ovaries (number of cystic follicles) and hyperandrogenism (testosterone concentration).

Bacterial Genus	No. of Cycles		No. of Cystic Follicles		Testosterone Concentration	
	Spearman ρ	*p* Value	Spearman ρ	*p* Value	Spearman ρ	*p* Value
*Blautia*	−0.6437	0.0071	0.6815	0.0036	0.5735	0.0202
*Dorea*	−0.5999	0.0140	0.5795	0.0187	0.3971	0.1278
*Clostridium*	−0.6211	0.0102	0.7140	0.0019	0.5853	0.0172
*Lactococcus*	−0.5365	0.0322	0.5277	0.0356	0.6000	0.0140
*Parabacteroides*	−0.1526	0.5726	0.5412	0.0304	0.3529	0.1800
*Ruminococcus*	0.5878	0.0166	−0.7036	0.0024	−0.2353	0.3804

Positive Spearman *ρ* values indicate positive correlations, and negative Spearman ρ values indicate negative correlations.

## Data Availability

Sequence data used in this study have been submitted to the Sequence Read Archive (SRA) with the accession number PRJNA755463 (available from 31 December 2021). ASV tables and the sequences of ASV can be provided upon request.

## Supplementary Materials

The following are available online at https://www.mdpi.com/article/10.3390/nu13113759/s1. Figure S1: stages of the rat menstrual cycle, Figure S2: alpha diversity analysis by Chao1 index, Figure S3: beta diversity analysis of C and L groups, Figure S4: relative abundance of microbial functional pathway responsible for bacterial invasion of epithelial cells (Bar chart), Figure S5: analysis of SCFAs, Figure S6: reproductive organ weights, Figure S7: metabolic syndrome parameters in serum samples, Table S1: soyaflavone HG (soy isoflavone supplement) composition, Table S2: weekly average body weight analysis.

## Abbreviations

ANOVA	analysis of variance
cDNA	complementary DNA
CMC	carboxymethyl cellulose
DEPC	diethylpyrocarbonate
DOGMA	dysbiosis of gut microbiota
EDTA	ethylenediaminetetraacetic acid
ELISA	enzyme-linked immunosorbent assay
FLASH	fast length adjustment of short reads
FSH	follicle stimulating hormone
GAPDH	glyceraldehyde 3-phosphate dehydrogenase
HDL	high-density lipoprotein
IBM	international business machines corporation
KEGG	Kyoto encyclopedia of genes and genomes
LDL	low-density lipoprotein
LH	luteinizing hormone
NaOH	sodium hydroxide
NGS	next-generation sequencing
OTU	operational taxonomic unit
PCOS	polycystic ovary syndrome
PCR	polymerase chain reaction
PICRUSt	phylogenetics investigation of communities by reconstruction of unobserved states
QIIME	quantitative insights into microbial ecology
qPCR	quantitative PCR
qRT-PCR	quantitative reverse transcription PCR
SCFA	short-chain fatty acids
SD	Sprague Dawley
SEM	standard error of measurement
SPF	specific pathogen free
SPSS	statistical package for the social sciences
STAMP	software testing amplification
UDGs	uracil-DNA glycosylases
