# Supplementary material for: Effects of Soy Isoflavones, Resistant Starch and Antibiotics on Polycystic Ovary Syndrome (PCOS)-Like Features in Letrozole-Treated Rats
Source: Nutrients. 2021 Oct 24;13(11):3759. doi: 10.3390/nu13113759 (PMC8621859; doi:10.3390/nu13113759)
Supplement: Supplementary file 1 [file nutrients-13-03759-s001.zip › nutrients-1405096-supplementary.pdf]

Supplementary Table S1: Soyaflavone HG (soy isoflavone supplement) composition

	Isoflavone content	Aglycone equivalent content
Daidzin	4.0%	2.4%
Glycitin	2.7%	1.7%
Genistin	0.3%	0.2%
Malonyl daidzin	28.8%	12.2%
Malonyl glycitin	10.1%	5.2%
Malonyl genistin	5.2%	2.3%
Acetyl daidzin	0.8%	0.5%
Acetyl glycitin	0.2%	0.1%
Acetyl genistin	0%	0%
**Total**	**52.1%**	**24.6%**

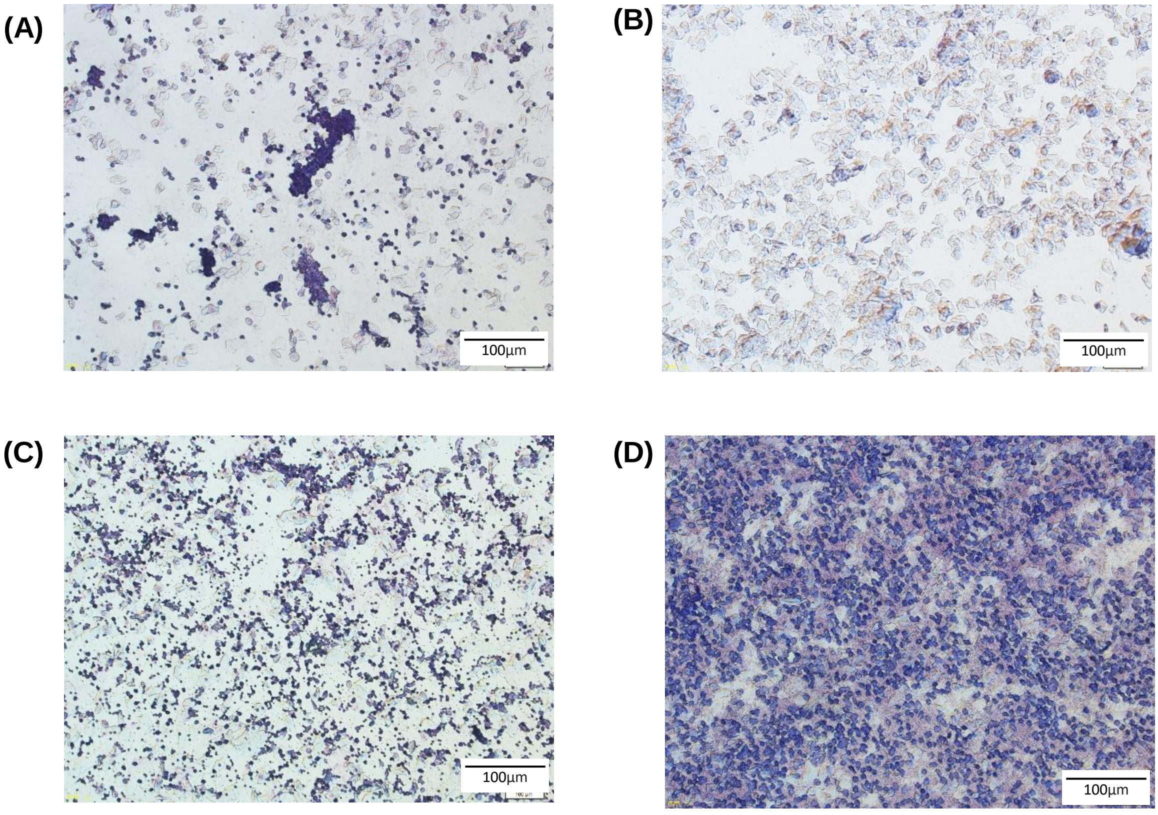

Supplementary Figure S1: Stages of the rat menstrual cycle. A normal female rat has four different stages in its menstrual cycle. (**A**) Proestrus stage has many small nucleated epithelial cells and their cohesive clusters. (**B**) Estrus stage has many cornified epithelial cells and some small and large nucleated epithelial cells. (**C**) Metestrus stage can be characterized by many neutrophils and cornified epithelial cells. (**D**) Diestrus stage also has many neutrophils entrapped in mucous, but none or a few cornified epithelial cells
Scale bars = 100 µm

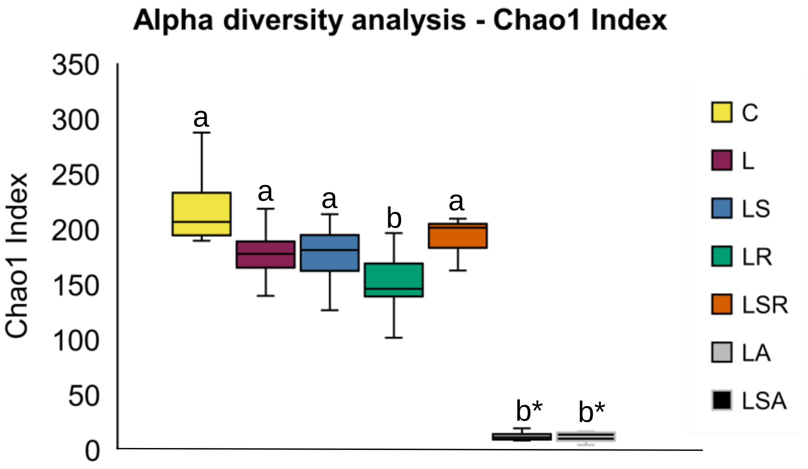

Supplementary Figure S2: Alpha diversity analysis by Chao1 index All the values are expressed as mean ± S.E. Statistical analysis was carried out by Kruskal-Wallis test followed by Tukey Kramer test for multiple comparison analysis. a, b represent $p < 0.05$ compared to C group, * represents $p < 0.05$ compared to L group

(A) Weighted beta diversity analysis

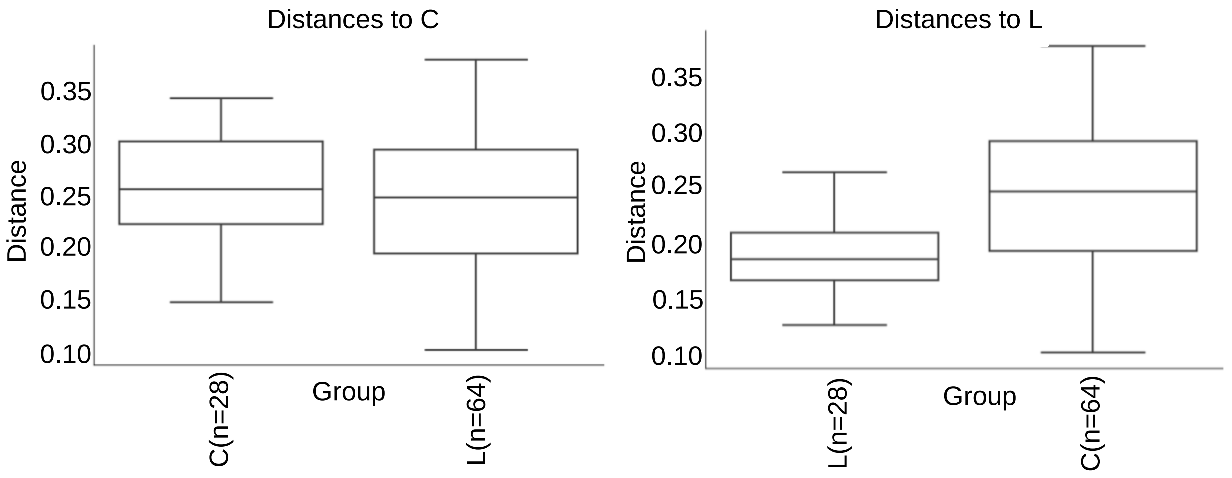

Group 1	Group 2	Sample size	Permutations	pseudo-F	p-value	q-value
**C**	**L**	16	999	2.203774	0.051	0.051

(B) Unweighted beta diversity analysis

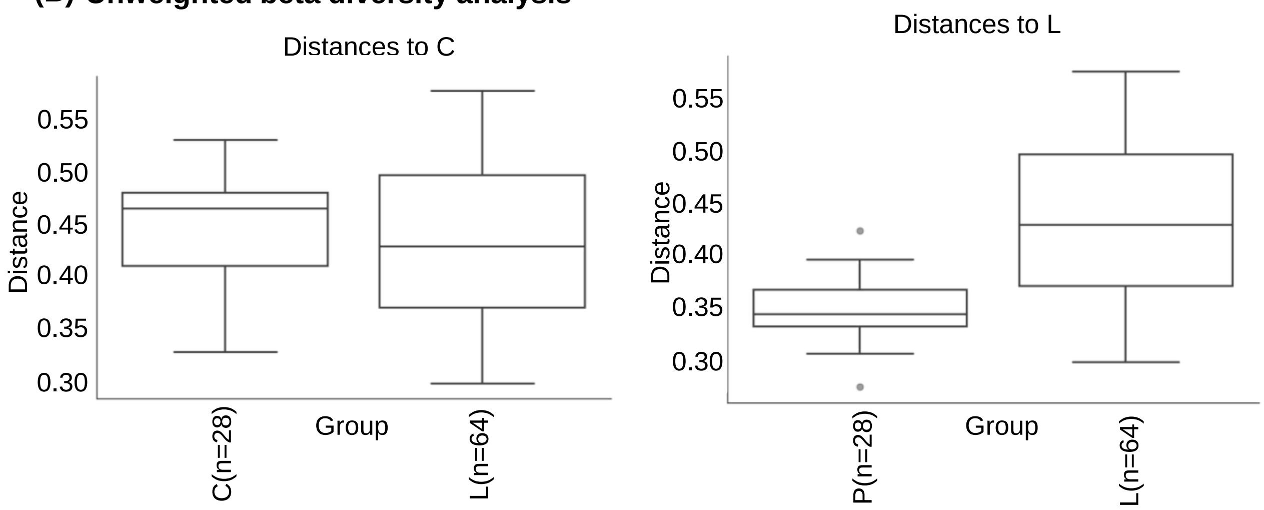

Group 1	Group 2	Sample size	Permutations	pseudo-F	p-value	q-value
**C**	**L**	16	999	2.921088	0.001	0.001

Supplementary Figure S3: Beta diversity analysis of C and L groups (**A**) Weighted and (**B**) Unweighted beta diversity analysis. All the values are expressed as mean ± S.E. Statistical analysis was carried out by Kruskal-Wallis test.

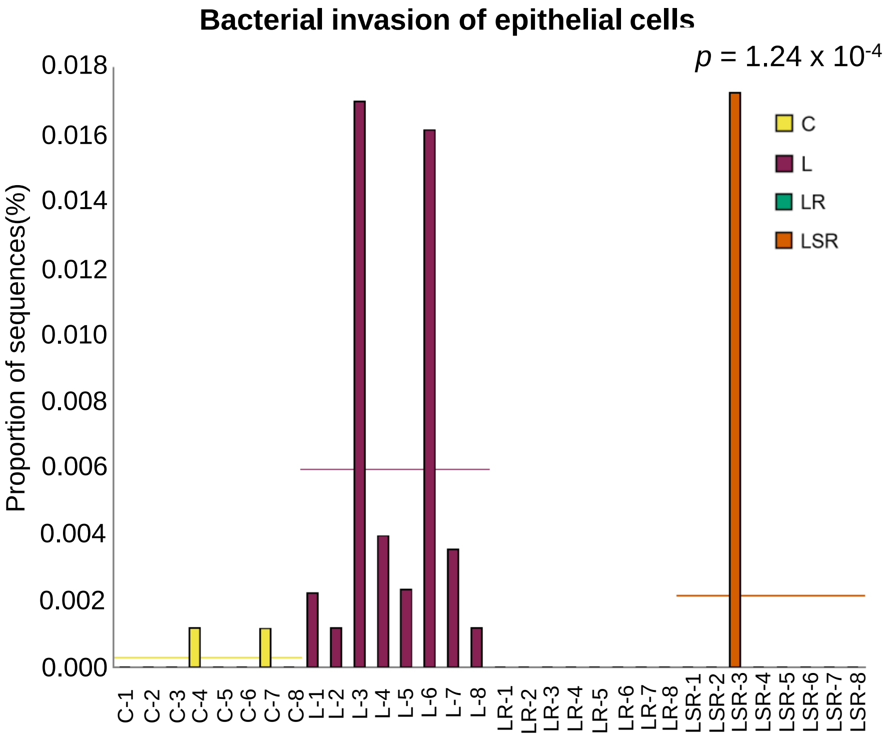

Supplementary Figure S4: Relative abundance of microbial functional pathway responsible for bacterial invasion of epithelial cells (Bar chart). All the values are expressed as mean ± S.E. Statistical analysis was carried out by Kruskal-Wallis test followed by Tukey Kramer test for multiple comparison analysis.

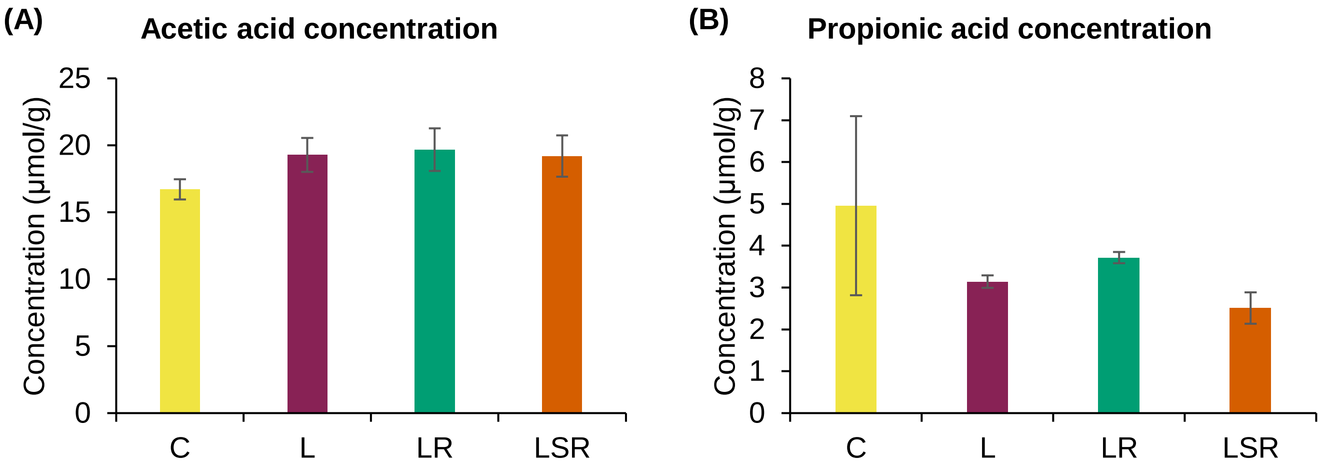

Supplementary Figure S5: Analysis of SCFAs. (**A**) Acetic acid concentration and (**B**) Propionic acid concentration. Concentrations of the SCFAs were measured using the plasma samples. All the values are expressed as mean ± S.E. Statistical analysis was carried out by one-way ANOVA followed by Dunnett's test for multiple comparison analysis.

Supplementary Table S2: Weekly average body weight analysis

Group	Acclimatization period	Pre-vaginal sampling	Letrozole treatment		
	Week 1	Week 2	Week 3	Week 4	Week 5
C	127.92 ± 1.97	166.71 ± 4.96	192.26 ± 5.19	217.98 ± 5.55^{a*}	239.59 ± 5.96^{a*}
L	128.28 ± 2.16	167.22 ± 4.67	199.29 ± 5.27	243.20 ± 6.70^{b}	282.23 ± 7.99^{b}
LS	127.30 ± 1.34	165.64 ± 3.98	196.44 ± 4.58	234.84 ± 5.68	273.50 ± 7.21^{b}
LR	125.22 ± 1.11	166.30 ± 3.39	199.37 ± 4.19	241.64 ± 4.36	275.51 ± 4.94^{b}
LSR	126.10 ± 2.05	165.52 ± 3.02	194.06 ± 4.11	230.27 ± 6.14	265.47 ± 7.30
LA	126.18 ± 1.22	166.65 ± 2.86	178.94 ± 4.33*	216.76 ± 5.49*	255.96 ± 5.83
LSA	123.97 ± 1.67	165.09 ± 3.49	175.97 ± 4.13*	213.15 ± 5.92*	247.77 ± 7.02*

All the values are expressed as mean ± S.E. Statistical analysis was carried out by one-way ANOVA followed by Dunnett's test for multiple comparison analysis. a, b represent $p < 0.05$ compared to C group, * $p < 0.05$ compared to P group.

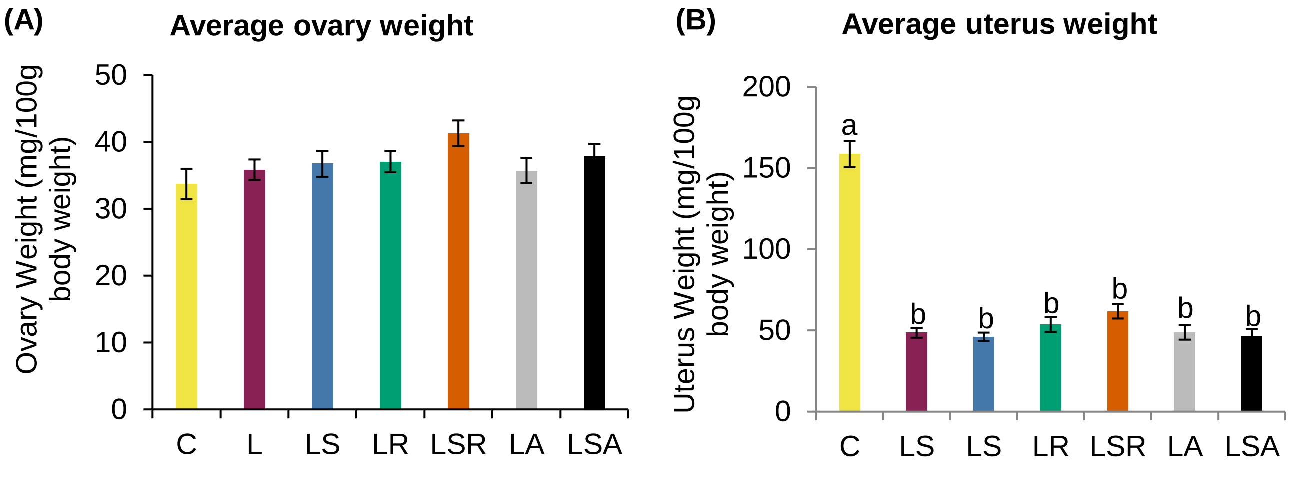

Supplementary Figure S6: Reproductive organ weights (**A**) Ovary weight (**B**) Uterus weight. All the values are expressed as mean ± S.E. Statistical analysis was carried out by one-way ANOVA followed by Dunnett's test for multiple comparison analysis. a, b represent $p < 0.05$ compared to C group

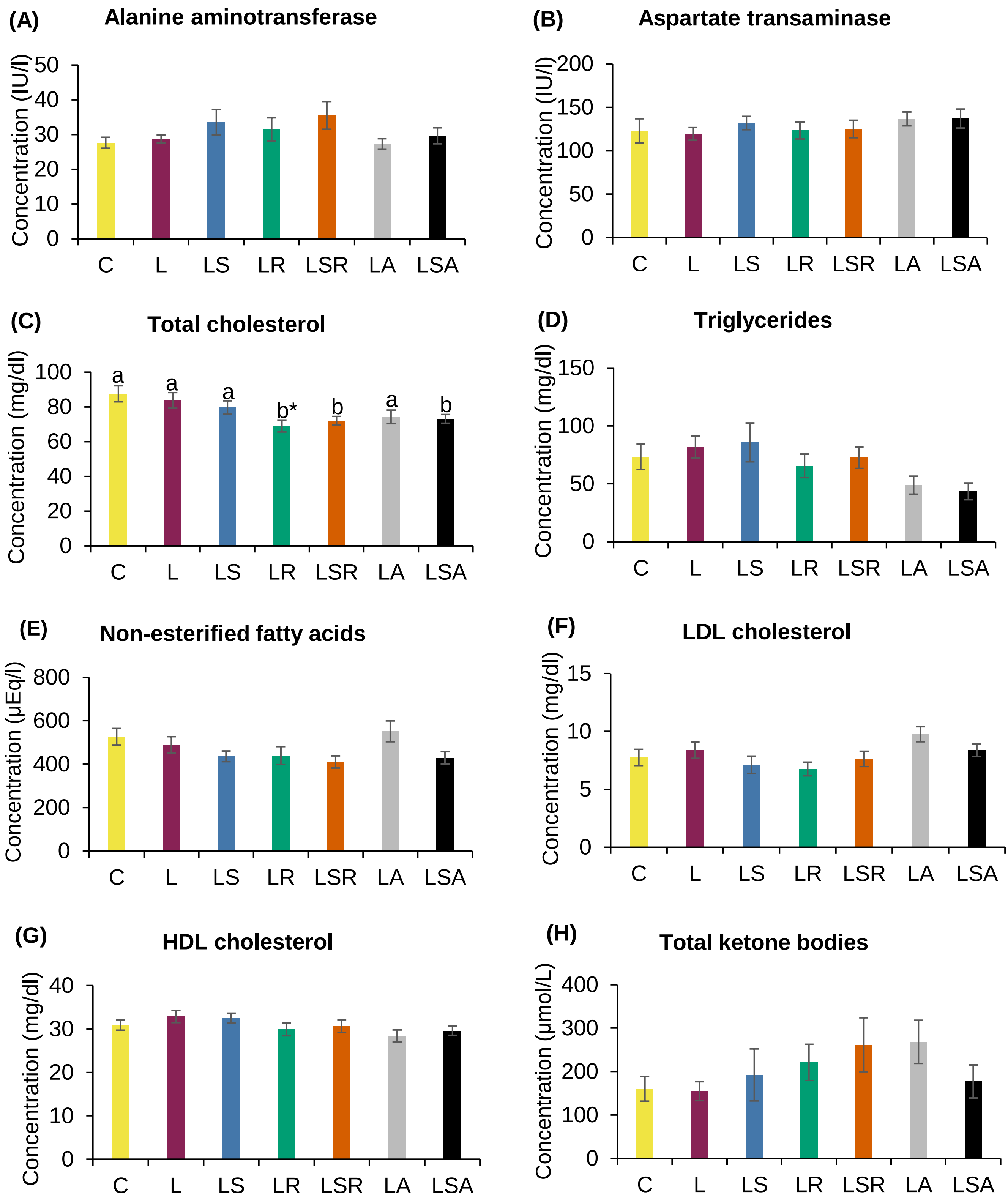

Supplementary Figure S7: Metabolic syndrome parameters in serum samples. (**A**) Alanine aminotransferase, (**B**) Aspartate transaminase, (**C**) Total cholesterol, (**D**) Triglycerides, (**E**) Non-esterified fatty acids, (**F**) LDL cholesterol, (**G**) HDL cholesterol and (**H**) Total ketone bodies. All the values are expressed as mean ± S.E. Statistical analysis was carried out by one-way ANOVA followed by Dunnett's test for multiple comparison analysis. a, b represent $p < 0.05$ compared to C group, * represents $p < 0.05$ compared to L group.
